# Withaferin A Suppresses Beta Amyloid in APP Expressing Cells: Studies for Tat and Cocaine Associated Neurological Dysfunctions

**DOI:** 10.3389/fnagi.2018.00291

**Published:** 2018-09-27

**Authors:** Sneham Tiwari, Venkata Subba Rao Atluri, Adriana Yndart Arias, Rahul Dev Jayant, Ajeet Kaushik, Jonathan Geiger, Madhavan N. Nair

**Affiliations:** ^1^Institute of NeuroImmune Pharmacology, Center for Personalized Nanomedicine, Department of Immunology, Herbert Wertheim College of Medicine, Florida International University, Miami, FL, United States; ^2^Biomedical Sciences, School of Medicine and Health Sciences, University of North Dakota, Grand Forks, ND, United States

**Keywords:** beta amyloid (Aβ), Withaferin A, HIV-1 Tat, cocaine, neurological disorders

## Abstract

Neurological disorders are the biggest concern globally. Out of ~36 million human immunodeficiency virus (HIV) positive people, about 30%–60% exhibit neurological disorders, including dementia and Alzheimer’s disease (AD) like pathology. In AD or AD like neurological disorders, the pathogenesis is mainly due to the abnormal accumulation of extracellular amyloid beta (Aβ). In this era of antiretroviral therapy, the life span of the HIV-infected individuals has increased leading towards increased neurocognitive dysfunction in nearly 30% of HIV-infected individuals, specifically older people. Deposition of the Aβ plaques in the CNS is one the major phenomenon happening in aging HIV patients. ART suppresses the viral replication, but the neurotoxic protein (Tat) is still produced and results in increased levels of Aβ. Furthermore, drugs of abuse like cocaine (coc) is known to induce the HIV associated neurocognitive disorders as well as the Aβ secretion. To target the Tat and coc induced Aβ secretion, we propose a potent bifunctional molecule Withaferin A (WA) which may act as a neuro-protectant against Aβ neurotoxicity. In this study, we show that WA reduces secreted Aβ and induced neurotoxicity in amyloid precursor protein (APP)-plasmid transfected SH-SY5Y cells (SH-APP). In this study, we show that in SH-APP cells, Aβ secretion is induced in the presence of HIV-1 Tat (neurotoxic) and drug of abuse coc. Our fluorescent microscopy studies show the increased concentration of Aβ40 in Tat (50 ng/ml) and coc (0.1 μM) treated SH-APP cells as compared to control. Our dose optimization study show, lower concentrations (0.5–2 μM) of WA significantly reduce the Aβ40 levels, without inducing cytotoxicity in the SH-APP cells. Additionally, WA reduces the Tat and cocaine induced Aβ levels. Therefore, we propose that Aβ aggregation is induced by the presence of Tat and coc and WA is potent in reducing the secreted Aβ and induced neurotoxicity. Our study provides new opportunities for exploring the pathophysiology and targeting the neurological disorders.

## Introduction

The overall life expectancy of people living with human immunodeficiency virus (HIV; People living with HIV, PLWH) has increased moderately due to introduction of effective anti-HIV therapies (Oguntibeju, 2012; Sabin, 2013). As per WHO Number of AIDS related death decreased from 1.5 million (2010) to 1.1 million (2015) globally (World Health Organization, 2016). Longer drug (anti-retroviral) consumption and virus living cycle leads to increased prevalence of HIV-1 associated neurocognitive disorder (HAND; Saylor et al., 2016). Additionally, PLWH (~2 million as per World Health Organization, 2018) are more prone to the risk of developing neurological diseases like Alzheimer’s disease and (AD)-like neurocognitive problems. HIV-infection and associated neurological disease synergism has become a pressing health issue to be managed, globally’ because HIV-infection progression facilitates AD like pathology (Koppel et al., 1985; Levy et al., 1985). Though, neurological disorders are irreversible but investigating novel therapies of better efficacy to manage these serious disorders without side-effects are urgently required.

AD is one of the prominent neurodegenerative disease, and is characterized as a progressive impairment of memory and neurocognitive functions due to abnormal accumulation of extracellular amyloid beta (Aβ) and intracellular neurofibrillary tangles (NFTs; Dorszewska et al., 2016). Aβ aggregation is prominent in the cortical and limbic regions of the brain (Snider et al., 1983; Kurapati et al., 2013, 2014). Alternative or abnormal cleavage of integral membrane amyloid precursor protein (APP) by β and γ secretases (Ghosh et al., 2008; Guardia-Laguarta et al., 2010) lead to abnormal Aβ processing, resulting into insoluble Aβ aggregation (Zheng et al., 2002; Kretner et al., 2016). Aβ peptides then aggregate into extracellular insoluble senile plaques (Echeverria et al., 2004; Guardia-Laguarta et al., 2010; Ahyayauch et al., 2012). This Aβ accumulation leads to decreased neuronal health and stability, increased deterioration, synaptic depression (Venkitaramani et al., 2007; Palop and Mucke, 2010; Li et al., 2017), oxidative stress (Butterfield et al., 2013; Arimon et al., 2015; Cheignon et al., 2018), augmented neuronal dysfunctions and inflammation (Barage and Sonawane, 2015; Marottoli et al., 2017). These dysfunctions caused by Aβ aggregation, become worst upon the presence of HIV-1 (András and Toborek, 2013; Martínez-Bonet et al., 2018) and drugs of abuse. HIV patients are reported to have augmented Aβ plaques deposition in the brain compared to HIV negative individuals (Esiri et al., 1998; Becker et al., 2004; Valcour et al., 2004). HIV associated Aβ dysfunction can be due to either the entire HIV virus, or mainly by neurotoxic Tat (transactivator of transcription) protein (Bagashev and Sawaya, 2013). HIV-1 Tat is neurotoxic and even though ART targets all the active virus, Tat could still be produced by the provirus in the viral reservoirs, such as brain (Daily et al., 2006). Tat protein as a neurotoxin, plays a prominent role in HIV neuropathogenesis as it gets secreted extracellularly and has the ability to cause neurotoxicity in the healthy cells (Chandra et al., 2005; Tahirov et al., 2010). Tat may have specific reaction with the Aβ in the CNS and facilitate Aβ aggregation, in the CNS (Hategan et al., 2017). Moreover, Aβ aggregations are studied to be increased in cortex of HIV brains when compared to age matched non-HIV controls (Achim et al., 2009; Soontornniyomkij et al., 2012).

Another factor, which augments the Aβ aggregation induced pathogenesis, are the drugs of abuse (Ramage et al., 2005; Dublin et al., 2017). These powerfully addictive stimulant drug molecules have been studied to have an exaggerating effect during HIV infection (Jayant et al., 2017). Cocaine (coc), a very common abused drug within PLWH, exerts malicious effects on the CNS (Javadi-Paydar et al., 2017; Meade et al., 2017; Wakim et al., 2017). In the presence of coc, the additive effect of HIV-1 Tat and coc may increase Aβ aggregation, which is a common factor in aging and HIV associated neurological disorders. Therefore, investigating desired therapies for coc abusing aging PLWH, are required for devising new therapeutic agent with multifunctional abilities to manage neurological disorders.

In this article, we have studied therapeutic properties of Withaferin A (WA) against multiple disease associated factors including Aβ, HIV-1 Tat and drug of abuse, coc. WA is an active purified drug moiety extracted from Ashwagandha (ASH), isolated from the root extract of a medicinal plant *Withania Somnifera* and expected to reverse Aβ_1–42_ induced toxicity in human neuronal cells (Kurapati et al., 2013, 2014). WA is a steroidal lactone, derived from *Withania somnifera* (Indian Winter cherry or Ashwagandha). ASH has been traditionally used in ayurvedic medicine. WA is the first member of the withanolide class of ergostane type product to be discovered (Mirjalili et al., 2009). The beneficial effects of WA has been studied in the field of tumor inhibition (Bargagna-Mohan et al., 2007), antiangiogenic activity (Mohan et al., 2004; Challa et al., 2012; Mohan and Bargagna-Mohan, 2016), and against angioproliferative and malignant diseases like pancreatic cancer (Yu et al., 2010), leukemia, breast cancer and colon cancer (Choi and Kim, 2015), as well as anti-metastasis (Lee and Choi, 2016) and anti-carcinogenic properties (Rah et al., 2012). However, the therapeutic ability of WA against neurological disorders, as a protective agent is not well studied yet. WA is also explored in the field of apoptosis and adipogenesis inhibitor in 3T3-L1 adipocytes (Park et al., 2008). In this systematic study, we have explored for the first time the neuroprotective role of WA against Aβ secretion and aggregation *in vitro*. During our study we observed the deleterious effect of Aβ on the neuronal health, function and morphology. In our step by step dose dependent studies, we explored the role of WA in reducing Aβ induced neurotoxicity in the HIV-1 Tat and coc treated APP-plasmid transfected SH-SY5Y cells (SH-APP) cells, towards neurological dysfunctions. The outcomes of this research claim that WA has a great potential to be promoted as a natural neuro therapeutic agent in order to manage age or viral infection associated neurological disorders. Our studies open new areas of drug efficacy against neurological conditions.

## Materials and Methods

### Chemicals and Reagents

Withaferin A (WA) was commercially purchased from Sigma Aldrich (Cat# W4394 SIGMA). Methylthiazolyldiphenyl-tetrazolium bromide (MTT; Cat# M2003) and paraformaldehyde was purchased from Sigma Aldrich. HIV-1 clade B recombinant Tat protein (86-amino acid) was obtained from NIH AIDS research and reference reagent program (Cat# 2222).

### Cell Culture

The cell type used in this study are SH-APP cells which is a human neuroblastoma cell line stably over-expressing human APP751 which was a kind gift from Dr. Jonathan Geiger (University of North Dakota, Grand Forks, ND, USA). SH-APP Cells were cultured in Dulbecco Eagle’s minimum essential medium (DMEM; Gibco^®^; Life Technologies, Grand Island, NY, USA) supplemented with 10% fetal bovine serum, 100 U/ml penicillin/streptomycin, nonessential amino acids, and sodium pyruvate (1 mM) at 37°C in 5% CO_2_.

### Cell Viability Assay

Cells were plated at a density of 1 × 10^4^ cells per well into 96-well plates and maintained at 37°C for 24 h. Cells were treated with various concentrations of WA for 48 h. Fresh medium containing 50μL of MTT solution (0.5 mg/mL) was added to each well. After 3 h incubation, the MTT formazan crystals were dissolved in dimethyl sulfoxide (DMSO) and viable cells were detected by measuring the absorbance at 570 nm using a microplate reader (Molecular Devices, Sunnyvale, CA, USA).

For Tat and coc toxicity study on cell viability, we performed Cell viability test using 0.4% Trypan Blue Solution (T8154) Live dead screening. Ten microliter of cells were taken from the pellet resuspended in fresh media, after centrifugation at 1,500 rpm for 5 min, and was mixed with 10 μl of Trypan blue dye (1:1 ratio). The cells were then loaded on a cell counting slide and counted for live count on a cell counter (BioRad TC20™ Automated cell counter).

#### Tat and Coc Treatment of SH-APP Cells

SH-APP cells (1 × 10^6^ cells) were cultured overnight in T-25 flasks in complete DMEM media with 10% FBS and 1% Penicillin streptomycin solution. After 48 h, cells were treated with different concentrations of HIV-1 Tat (5–100 ng/ml) and coc (0.1–10 μM) and the cells and supernatant were collected after 48 h after the treatment. The optimized dose of Tat and coc were selected based on their effect on increasing Aβ levels significantly compared to untreated controls. In further experiments, 1 × 10^5^ SH-APP cells were seeded in six wells plates and were cultured for 48 h. Cells were treated with optimized concentrations of HIV-1 Tat1–72 and/or coc.

#### Quantification of Aβ40 Levels

Secreted Aβ levels were measured using human Aβ40 ELISA kit as per the manufacturer’s protocol (Thermo Fisher Scientific, Catalog# KHB3481). For secreted Aβ measurements, SH-APP cells were cultured in six well plates and after 48 h, cells were treated with HIV-1 Tat/coc in combination with WA. The media from cultured cells was collected and protease inhibitor was added to it. The supernatant was utilized as samples for the AB_40_ ELISA as per the specific reagents and protocol provided with the kit. Each sample was analyzed in duplicate. Cells were saved for flow cytometry studies to estimate intracellular Aβ40 level.

#### Flow Cytometry

Cells from Tat, coc^+/–^ WA treated samples were utilized for flow cytometry studies. Flow Cytometry was used to identify the expression of Aβ40 protein in SH-APP cells after treatment with various concentrations of Withaferin A, Tat and coc. 1 × 10^6^ SH-APP cells were stained with primary anti-human Aβ40 (#PA3–16760) and secondary anti-rabbit Fluorescein isothiocyanate (FITC)-labeled antibody (catalog #AP187F, Millipore). Auto fluorescence of the cells was based on the unstained cells. Cells were gated based on the secondary antibody. Accuri BD flow and Amnis^®^ Imaging Flow Cytometers were used for acquisition. Analysis was conducted in Flow Jo software and Amnis^®^ FlowSight^®^ Imaging Flow Cytometer and analysis by IDEAS^®^ image software.

### Single-Cell Flow Cytometry

The SH-APP were treated with different concentrations of WA. The cells were then harvested at 24 h after treatment, washed and counted; equal amounts of cells (1× 106) were aliquoted in 1.5 ml Eppendorf centrifuge tubes in 250 μl 1× PBS. Cells were analyzed by ImageStreamX Imaging Flow Cytometer (Amnis Corporation, Seattle, WA, USA) having with INSPIRE software. A magnification of 60× was employed for all readings. Ten-thousand cells (events) were analyzed for each sample. FITC and DAPI were excited with a 100 mW of 488 nm argon laser. FITC and DAPI fluorescence was collected on channel two (505–560 nm) and channel seven (560–595 nm), respectively. Intensity adjusted bright field images were collected on channel one. Bright field area and total fluorescence intensity were calculated using IDEAS software. Data analysis was performed using the IDEAS software (Amnis Corporation), with proper data compensation with respect to singly stained samples. The compensated data was then gated to eliminate cells that were out of field of focus and doublets or debris was eliminated too.

#### Immunofluorescence Staining and Analysis for Studying Beta Amyloid Aggregation

To study the effect of WA on the morphology aggregation, we conducted immunofluorescence imaging experiment. The cells were cultured to 80% confluency on the 4-well microscopy slides and were then exposed to HIV-1 Tat+/− WA. After 48 h, the supernatant was discarded and the cells were fixed in 4% PFA. PFA embedded slides were then immunostained by using Aβ40 primary antibody (1:100) and GFP secondary antibody (1:100). Immunohistochemically stained sections were captured using the Keyence microscope. The images were captured at a magnification of 10×.

#### Immunofluorescence Staining for Studying Effect of WA on Neuronal Morphology

To study the effect of WA on the neuronal morphology, we conducted immunofluorescence imaging experiment. The cells were cultured to 80% confluency on the 4-well microscopy slides and were then treated with HIV-1 Tat protein/coc +/− WA. After 48 h, the supernatant was discarded and the cells were fixed in 4% PFA. PFA embedded slides were then washed and immunostained using MAP2 primary antibody (1:100) and anti-FITC secondary antibody (1:100). Immunohistochemically stained sections were captured using the ImageScope AT2 image scanner (Aperio Technologies) and analyzed using the ImageScope software; Scale 50 μm.

### Data Analysis

Results in this study are representative of three or more independent experiments. Statistical significance was analyzed using Graph Pad Prism5 software, La Jolla, CA, USA by performing ANOVA or the Student’s *t*-test for unpaired observations. The values are presented as means ± SEM.

## Results

### WA Dose Optimization and Aβ Neutralizing Efficacy Studies in SH-APP Cells

**Figure 1:**
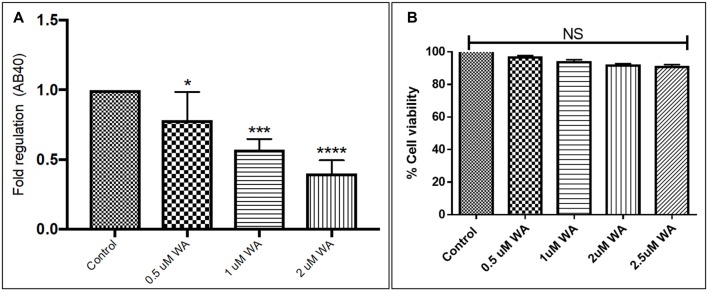
Effect of Withaferin A (WA) on amyloid beta (Aβ) secretion. **(A)** Cells were treated with different dose of WA and the supernatants were collected 48 h after treatment. The supernatant collected were analyzed by Aβ_1–40_ ELISA which demonstrated that at an optimum dose of 2 μM WA, the levels of secreted Aβ40 showed significant decrease compared to control untreated samples. **(B)** The dosage of WA used for this experiment were also analyzed for the associated cellular toxicity. The cell toxicity assay showed that the lower doses of WA were not toxic to cells. Optimum dose of 2 μM, WA did not cause any loss in cell viability or toxicity in amyloid precursor protein (APP)-plasmid transfected SH-SY5Y (SH-APP) cells (*p ≤ 0.05; ***p ≤ 0.001; ****p ≤ 0.0001).

**Figure 2:**
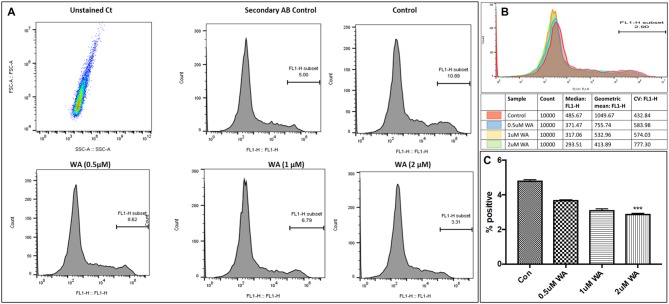
WA inhibits Aβ_1–40_ in concentration dependent manner. Panel **(A)** shows histograms of Aβ secretion by the SH-APP cells upon treatment with varying dose of WA. Panel **(B)** shows the layover of the peaks in one histogram, and **(C)** shows the quantification of the same. The cells were treated with WA concentrations, and after 48 h of treatment were analyzed by Flow cytometry for determining Aβ_1–40_ levels. Flow cytometry was used to identify the expression of Aβ_1–40_ in SH-APP cells after treatment with three different concentration of WA. 1× 106 SH-APP cells were stained with primary anti-human Aβ40 (#PA3–16760) and secondary anti-rabbit Fluorescein isothiocyanate (FITC)-labeled antibody (catalog #AP187F, Millipore). Auto fluorescence of the cells was based on the unstained cells. Cells were gated based on the secondary antibody. Accuri BD flow and Amnis^®^ Imaging Flow Cytometers were used for acquisition. Analysis was conducted in Flow Jo software and Amnis^®^ FlowSight^®^ Imaging Flow Cytometer and analysis by IDEAS^®^ Image software. For each experiment, from all events collected, FITC positive cells were gated from single cells (***p ≤ 0.001).

**Figure 3:**
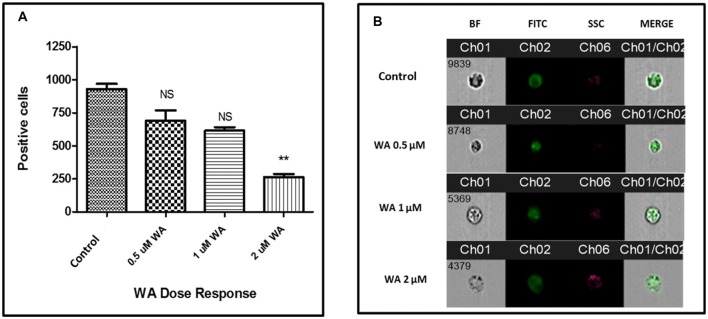
WA inhibits Aβ production: single cell flow Cytometry was used to identify the expression of Aβ_1–40_ protein in SH-APP cells after treatment with different concentration of WA. **(A)** Bar graph representing the mean ± standard error of percent of mean fluorescence intensity. **(B)** Representative single cell images. We have observed significantly reduced Aβ with WA exposure dose dependently (*n* = 3; **p ≤ 0.01; NS: Non Significant).

To optimize the non-toxic dose of WA, different concentrations of WA (0.5–10 μM) were treated to SH-APP (neuroblastoma cell lines stably expressing human APP751) cells and results showed that 2 μM of WA reduces the secreted Aβ40 in SH-APP cells significantly when compared to non-treated control, (Figure 1A) without inducing cytotoxicity to the cells (Figure 1B). Further, results were confirmed with the flow cytometry and showed (Figures 2A–C) dose dependent reduction in the Aβ_1–40_ levels and the maximum reduction was reported at 2 μM WA concentration without causing cellular toxicity. Additional single cell flow cytometry and imaging also showed the same trend highlighting the effective role of 2 μM WA against Aβ_1–40_ (Figures 3A,B).

### Effect of HIV-Tat Protein and coc on Aβ Production in SH-APP Cells

**Figure 4:**
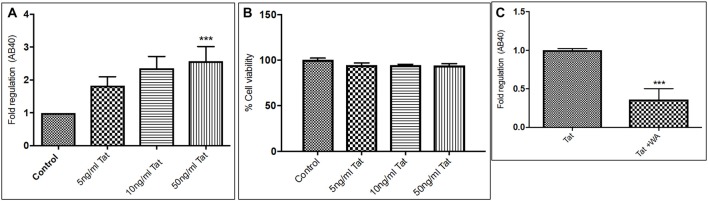
Tat induce increase in secreted Aβ40 levels **(A)**. Human Amyloid Beta ELISA analysis showing that Tat (5–50 ng/mL) increased the secreted Aβ_1–40_ significantly in SH-APP cells. **(B)** Cellular toxicity assay showing viability of the cells in the Tat treated samples. **(C)** 2μM WA reduced the Tat levels significantly when compared to Tat (50 ng/mL) only treated samples. 1× 106 SH-APP cells were seeded in 6-well plates and were grown for 48 h and then treated with human immunodeficiency virus (HIV)-1 Tat in different doses and the cells were then incubated for 48 h at 37°C. The supernatant from the culture was collected and treated with protease inhibitor (1μl/ml) and analyzed by Aβ_1–40_ ELISA (Sigma). The results are from three independent experiments and the statistical significance was calculated by Student’s *t*-test. Cell viability study was performed by Trypan blue live dead screening, to study the toxicity levels of various Tat dose. Dose selected for Tat treatment for further experiment was elected on the basis of increase in Aβ40 secretion levels and correlated with cell viability (***p ≤ 0.001).

**Figure 5:**
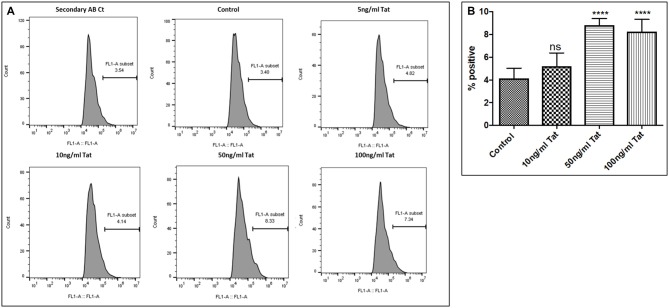
Dose response. **(A)** Histograms showing Tat (5–100 ng/mL) increases the Aβ_1–40_ levels. SHAPP cells were treated with different concentrations of Tat and after 48 h of treatment were analyzed by Flow cytometry for determining the Aβ_1–40_ levels. **(B)** Quantification representation of the percent positive cells (****p ≤0.0001; ns: Non Significant).

**Figure 6:**
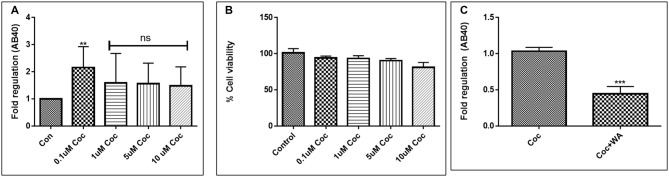
Coc induces increase in secreted Aβ40 levels. Similar study pattern of ELISA and flow cytometry, like in the case of HIV-1 Tat, was performed with various concentrations of coc to choose an optimized dose of coc for further studies. **(A)** Coc increases Aβ_1–40_ secretion. Coc (0.1–10 μM) increased the secreted Aβ_1–40_ but the significant increase was found in the samples treated with 0.1 μM coc. **(B)** Cellular toxicity assay showing viability of the cells in the coc treated samples. **(C)** 2μM WA reduced the coc (0.1 μM) induced Aβ_1–40_ levels significantly when compared to coc only treated samples (**p ≤ 0.01; ***p ≤ 0.001; ns, not significant).

**Figure 7:**
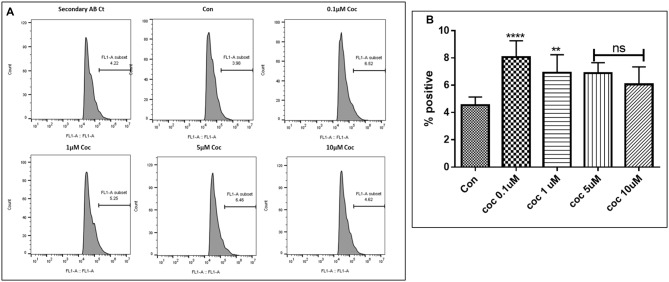
Dose response. **(A)** Histograms showing coc (0.1–10 μM) increases the Aβ_1–40_ levels. The cells were treated with different concentrations of coc and after 48 h of treatment were analyzed by flow cytometry for determining the Aβ_1–40_ levels. **(B)** Quantification representation of the percent positive cells (**p ≤ 0.01; ****p ≤ 0.0001; ns, not significant).

Human Aβ40 ELISA was performed with the supernatant collected from control and WA treated SH-APP cell samples in order to evaluate the efficacy of WA in reducing the HIV-Tat and coc induced Aβ secretion. SH-APP cells were treated with different concentrations of Tat (5–50 ng/ml) and coc (0.1–10 μM). Figure 4 shows that the SH-APP cells treated with Tat exhibited upregulation of Aβ_1–40_ secretion compared to untreated control (Figures 4A,B). Effective dose of Tat (50 ng/ml) when treated with 2 μM WA, showed significant decrease in Aβ_1–40_ (Figure 4C). Further, the results were also confirmed by the flow cytometry using Aβ_1–40_ specific primary antibody. The dose of 50 ng/ml Tat most significantly increased the Aβ levels when compared to control (Figures 5A,B). Additionally, we studied the effect of coc in the similar study pattern, and observed the increase in Aβ_1–40_ secretion (Figures 6A,B). We report that 0.1 μM showed most significant upregulation in Aβ_1–40_ secretion compared to untreated controls. Effective dose of coc (0.1 μM) when treated with 2 μM WA, showed significant decrease in Aβ_1–40_ (Figure 6C). This trend was also confirmed by the flow cytometry experiment which showed a coc induced increase in Aβ levels (Figures 7A,B).

### Tat and coc Induced Increase in Aβ40 Levels, in Combination

**Figure 8:**
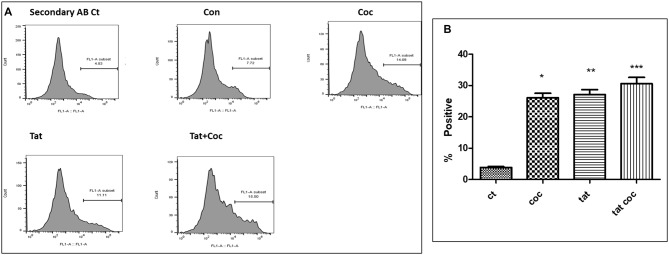
Tat and coc combination. **(A)** Tat (50 ng/mL) and coc (0.1 μM) individually and in combination increase the Aβ_1–40_ levels. The cells were treated with selected Tat and coc concentrations and combination of both, and after 48 h of treatment were analyzed by flow cytometry for determining the Aβ_1–40_ levels. **(B)** Quantification representation of the percent positive cells (*p ≤ 0.05; **p ≤ 0.01; ***p ≤ 0.001).

An optimized dose of Tat (50 ng/mL) and coc (0.1 μM) alone or in combination were used to study the neutralizing efficacy of WA (2 μm) in SH-APP cells. Results showed the combined effect of Tat and coc together in increasing the Aβ_1–40_ levels. (Figures 8A,B). Individual optimized dose of Tat (50 ng/mL) and coc (0.1 μM) were used for further WA neutralizing efficacy studies from here on.

### WA Reverses Tat and coc Induced Amyloid Aggregates *in vitro*

**Figure 9:**
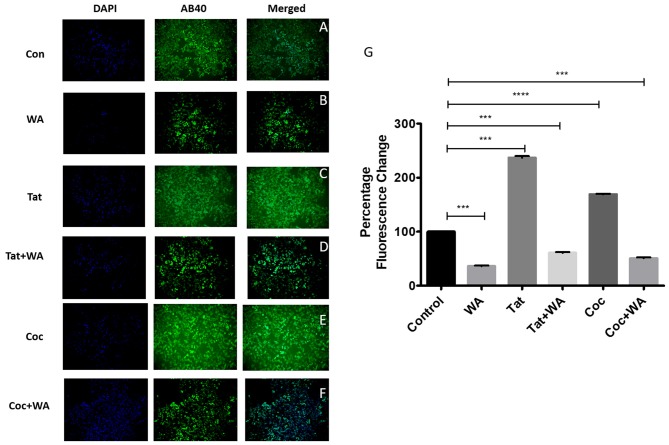
WA inhibits HIV-1 Tat induced Aβ-production, respectively. SH-APP cells were treated with HIV-1 Tat (50 ng/mL; **C**) coc (0.1 μM; **E**) +/− WA (2 μM; **(D,F)** respectively) were compared to Control **(A)** and only WA treated cells **(B)**. After 24 h, cells were fixed and stained with Anti-Human Aβ40 overnight. Cells were washed and stained with secondary anti-rabbit FITC-labeled antibody (catalog #AP187F, Millipore). Images were acquired using Keyence ALL in one microscope (10×). WA significantly suppressed Tat and coc induced Aβ-secretion, respectively (*n* = 3). Florescent intensity of these stained cells was quantified using the ImageJ software (**G**; ***p ≤ 0.001; ****p ≤0.0001).

Immunocytochemistry studies showed that WA was able to reduce the amyloid aggregation when compared to the untreated control SH-APP cells. The cells were grown in the microscopic slides (eight wells) and after 24 h of growth, the wells were treated individually with Tat+/− WA and coc+/− WA for 48 h and control wells had fresh media added. The cells were then collected, fixed and stained with primary Beta amyloid 1–40 antibody (1:100) and GFP secondary antibody (1:100). We observed that the cells exposed to Tat and coc had strong signals for amyloid beta aggregations, which was mitigated by WA treatment as seen in the Tat + WA and coc + WA microscopic chambers, when compared to control well (Figures 9A–G).

### WA Reverses/Decreases coc Induced Neurotoxicity

**Figure 10:**
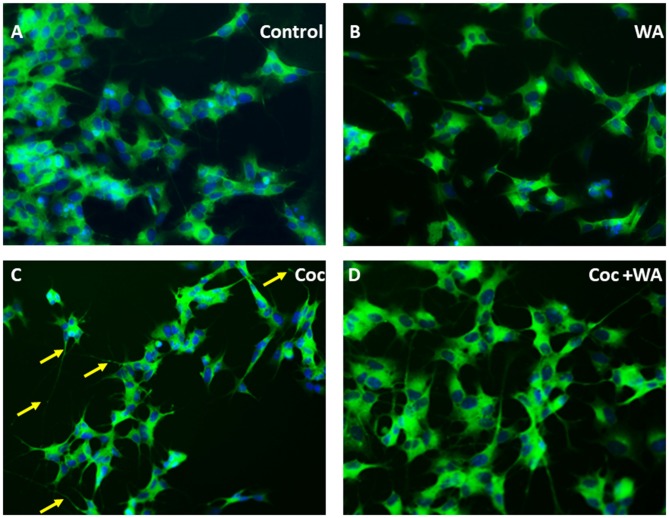
WA reverses coc induced dendritic beading and cytoplasmic vacuoles. SH-APP cells were treated with coc (0.1 μM) +/− WA (2 μM). After 24 h, cells were fixed and stained with MAP2 primary antibody overnight. Cells were washed and stained with secondary anti-rabbit FITC-labeled antibody (catalog #AP187F, Millipore). **(A)** Control SH-APP cells and **(B)** WA only treated cells showed no abnormal beading or thickening of the dendrites when compared to **(C)** coc exposed SH-APP cells which exhibited heavy dendritic beading (yellow arrows) and cytoplasmic vacuoles, measure of the cells being in drug-induced stress, **(D)** WA treated coc exposed cells on the other HIV-1 associated neurocognitive disorder (HAND) displayed reduced dendritic beading and elongated dendrites, with minimal cytoplasmic vacuoles.

Additionally, to study derogatory effects of coc on the SH-APP cells and the effect of WA on the neuronal morphology, we conducted immunofluorescence imaging experiment. Cultures of SH-APP cells grown on the eight well imaging slides for 48 h, and then the cells were stained by MAP2 primary antibody. We observed that the cells exposed to coc for 48 h exhibited heavy dendritic beading (indicated by yellow arrows) and cytoplasmic vacuoles (Figure 10C). The control SH-APP cells (Figure 10A) and WA only treated cells (Figure 10B) showed no abnormal beading or thickening of the dendrites, when compared. Upon treatment with WA, in coc exposed cells (Figure 10D), we observed reduced dendritic beading and more pronounced and elongated dendrites, communicating with other neuronal cells. We also observed reduced cytoplasmic vacuoles in the WA treated cells. This indicates that coc induces the stressed environment in the cell culture system which leads to neuronal damage (Figure 10).

## Discussion

Currently, the studies focusing towards neurodegeneration caused by either aging or due to viral infections, are extremely important. The accumulation of Aβ in the CNS is major factor contributing towards neurodegeneration (Green et al., 2005). The introduction of HAART gives a longer life span, giving a major opportunity to age related disorders in these recovering patients (Ellis et al., 2010; Heaton et al., 2010, 2011). The currently available drugs against Aβ aggregation, for example, Memantine (*N*-methyl-D-aspartic acid (NMDA) receptor antagonist), helps in repair of damaged neurons (van Marum, 2008), but does not aid in overall cure for neurological issues. Another drug which is very well studied for its anti-inflammatory, antioxidant and neuroprotective properties is Cucurmin/Curcuminoid, obtained from the roots of a plant Curcuma longa (Sharma et al., 2007). It has been reported that Curcuma may have potential role in AD treatment by targeting Aβ aggregates and associated toxicity in the neuronal cells (Ringman et al., 2005; Ishrat et al., 2009). Unfortunately, Curcuma is weakly stable and easily hydrolyzed, and gets photodegraded or even oxidized. This makes it very challenging and leads to its minimal bioavailability in the CNS (Anand et al., 2007).

Currently, there is no direct cure available for AD or AD-like neurodegenerative symptoms. Therefore in this paper we have focused on a drug compound WA, as a neuroprotective agent against Aβ induced neuronal toxicity. Our studies show that WA reduces the levels of secreted Aβ significantly without causing cytotoxicity in the cell cultures. Our microscopic studies demonstrate the protective role of WA as the human neuroblastoma cells showed healthy growth in the presence of WA. We observed that WA treatment reduced dendritic beading and cytoplasmic vacuoles in the SH-APP cells, conferring towards protective role of WA. Our observation coincides with other studies as well, which show that *W. somnifera* whole root extract treatment promotes neuronal health by inducing dendrite formation *in vitro* (Tohda et al., 2000; Zhao et al., 2002). Moreover, our study shows for the first time that a small sized active moiety of Withania root extract, termed as WA neutralizes secreted Aβ, in the SH-APP cells *in vitro*. Our previous lab has studied the role of Ashwagandha (ASH) which is a big molecule extracted from roots of *W. Somnifera*. We reported the properties of ASH towards neutralizing Aβ in the neuronal cells *in vitro*. ASH showed the reduction of Aβ in treated cells significantly when compared to untreated controls, suggesting anti-amyloid role of ASH (Kurapati et al., 2013, 2014). Even though ASH is capable of reducing the secreted Aβ, the understanding of ASH’s efficacy in the CNS across the BBB is minimum, as it is a big moiety. Therefore, the systematic delivery of the drug into the CNS and increased bioavailability becomes a pressing issue. This urged a need to find potent smaller molecular weight molecules with similar properties. Systematic chromatographic studies show the various components, upon breakdown of ASH molecule. This gave us an opportunity to study small molecule WA and assess its ability as a neuroprotectant to target the Aβ levels.

We further wanted to explore the effect of WA on induced Aβ production by the exposure of HIV-1 Tat. Therefore, in this study, we have analyzed the effect of HIV-1 Tat protein (Nuovo et al., 1994; Nath et al., 1996; Merino et al., 2011) on the Aβ secretion in SH-APP neuronal cells and found significantly increased Aβ production. Our results are in agreement with other studies which have reported the role of Tat protein in increased neuronal Aβ secretion (Rempel and Pulliam, 2005; Giunta et al., 2009; Aksenov et al., 2010). Tat is a neurotoxin and we show that it aggravates the Aβ aggregation *in vitro*. The mechanism through which this happens is still not understood well (Chen et al., 2013; Hategan et al., 2017), Tat may have a direct interaction with the Aβ fibrils, resulting in induced aggregation of monomers, towards plaques. This hypothesis is supported by our Immunocytochemistry studies which show dense accumulation of Aβ, in the cell medium exposed to HIV-1Tat (50 ng/ml). Our human Aβ40 ELISA experiment detected increased concentration of Aβ in Tat treated samples as well. This leads us to a conclusion that Tat is extremely neurotoxic and has an ability to interact with Aβ, increasing the overall toxicity of the cell system, and urging more and more release and aggravation of Aβ. HIV-1 Tat tends to have a direct physical interaction with Aβ peptide, leading to excessive aggregation of Aβ leading to neurotoxicity (Hategan et al., 2017).

Further, among the most abused drugs by People living with HIV (PLWH), coc abuse has been one of the major contributors towards the increased severity of neurocognitive disorders in the patients (Fiala et al., 1998; Gannon et al., 2011; Buch et al., 2012). Additionally, the percentage of drug abusers in the HIV positive population and aging population is very high. Drug abuse/addiction and HIV/AIDS are linked since the beginning of the HIV/AIDS epidemic. People who inject drugs accounted for about six percent of HIV diagnoses in 2015 (CDC, 2018). Even though the association of coc is shown with the exaggeration in HIV neuropathogenesis, the underlying mechanisms remain unclear. We elucidate the mechanism, in this study, for the first time, we observed the increased levels of Aβ production by coc. We also observed in our Immunocytochemistry experiments that coc affects neuronal morphology and communications, and aggregation of Aβ in the SH-APP cells, *in vitro*. This verifies the toxic effect of coc on the neuronal cells, which contribute in the increased accumulation of the amyloids. Coc alone and in combination with HIV-1 Tat is highly neurotoxic. These results coincide with various *in vivo* studies done by other research groups which show that the peritoneal injection of coc in rats stimulates hyperphosphorylation of tau and neurofilament in cortex, hippocampus and caudato-putamen regions of brain, indirectly contributing to the Aβ toxicity (Liu et al., 2003). These observations indicate that coc addiction may be associated with neurofibrillary degeneration. Therefore, here we report that coc in addition to HIV-1 Tat increases Aβ secretion *in vitro*. Our findings suggest that HIV-1 Tat and coc introduce cellular toxicity and cause neuronal dysfunctions by increasing amyloid secretion and modulating neuronal morphology and communications. Moreover, accumulation and deposition of Aβ in the brain of HIV patients (active infection or latent infection) drive the pathogenic cascades of neurological disorders, contributing towards aging or associated dementias (Pulliam, 2009). Targeting Aβ secretion, will have a translational significance in the treatment of HIV coc abusers and other neurological disorders like AD.

Additionally, the main rationale behind introducing WA is the unavailability of the direct medicine/drugs which target neurological disorders. Moreover the drugs, get rejected due to pharmacotherapy failures like inadequate physical chemistry, minimal absorption, unfavorable pharmacokinetic parameters, instability and toxicity. This urges the need of the alternate medicine/nanomedicine. Therefore, our next step is to address the limitation or incapability of drugs to cross BBB into the CNS, by employing nanotechnology assisted approaches, where our developed drug magneto-liposomes (which are a biopolymeric vesicle with capacity to deliver drugs across BBB) would be able to transmigrate across BBB (Ding et al., 2014; Kaushik et al., 2016).

In summary, it is critical to design and identify compounds that specifically target and inhibit Aβ secretion and aggregation, and also the interaction between Aβ and HIV-Tat 1 and drug of abuse, against their synergistic role towards neurodegenerative disorders. When combined with other strategies targeting Aβ, including immunotherapy, these approaches might allow for a reduction, if not elimination, of Aβ-related toxicity. Further *in vivo* efficacy and drug delivery mechanistic studies are necessary to explore WA’s therapeutic role in neurological disorders like HIV associated neurocognitive disorders and Alzheimer’s disease.

## Author Contributions

ST designed and coordinated the research, performed experiments, data analysis and manuscript writing. AYA helped in flow cytometry experiments and analysis. VA and RJ helped in data analysis and manuscript review. AK helped in manuscript review. JG reviewed the manuscript. MN guided in experimental research plan and provided continuous supervision and reviewed the manuscript.

## Conflict of Interest Statement

The authors declare that the research was conducted in the absence of any commercial or financial relationships that could be construed as a potential conflict of interest.
